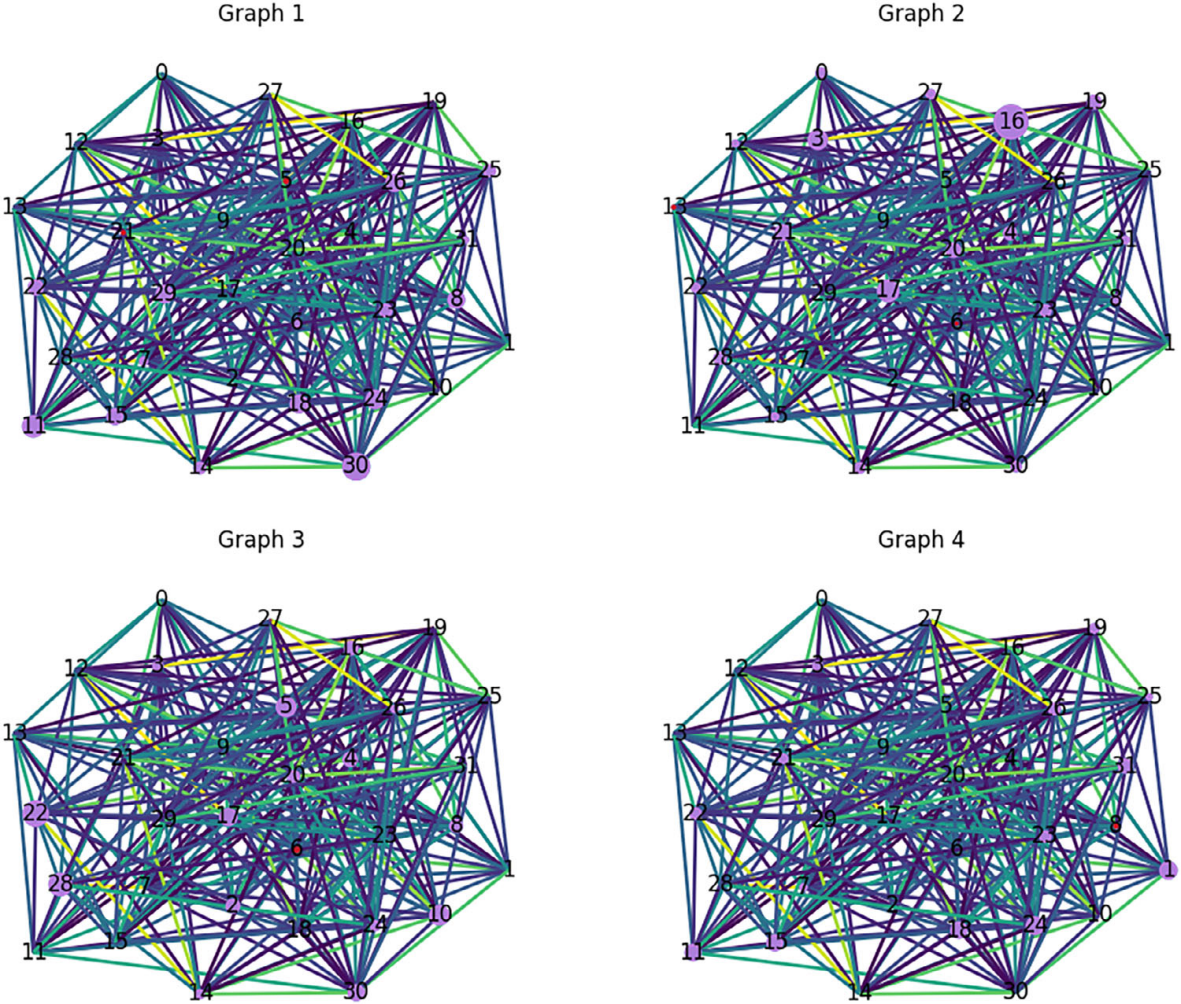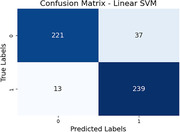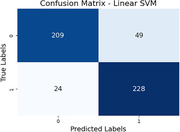# A Graph‐Theoretic Approach for fMRI Time‐Series Classification into Healthy and MCI

**DOI:** 10.1002/alz.085345

**Published:** 2025-01-09

**Authors:** Ameiy Acharya, Neelam Sinha

**Affiliations:** ^1^ Vellore Institute of Technology, Chennai, Tamil Nadu India; ^2^ Centre for Brain Research (CBR), Indian Institute of Science, Bengaluru, Karnataka India

## Abstract

**Background:**

Mild Cognitive Impairment (MCI), a transitional stage between healthy aging and Alzheimer's disease, offers a critical intervention window (Vega and Newhouse, 2014). Diagnosing MCI proves challenging due to subtle neurodegenerative changes. Our study employs graph representations of fMRI time‐series, emphasizing connectivity between brain regions (Stam and Reijneveld, 2007). Utilizing the Dosenbach‐160 ROI atlas and extracting eigenvalues and eigenvectors, we apply machine learning for classification.

**Method:**

Each subject contributes fMRI data from six networks, with each network comprising 187 timestamps and varying numbers of Regions of Interest (ROIs). For every network of each subject, we segment the timestamps into 11‐time interval batches, generating 17 graphs to encapsulate the subject's representation within a network. The principle guiding our approach is that similar eigenvalues reflect similar graphs. Construction of partial correlation graphs involves deriving precision matrices, where ROIs become graph nodes, and partial correlations form edge weights. Transforming these graphs into adjacency, degree, and Laplacian matrices, we further apply eigen‐decomposition on the Laplacian matrix to extract eigenvalues and eigenvectors. The eigenvectors corresponding to the largest absolute eigenvalue serve as features for SVM and Logistic Regression classification.

**Result:**

In our balanced dataset of 100 subjects (50 healthy, 50 MCI) sourced from publicly available ADNI database, SVM achieves accuracies of 80.39% (DMN), 76.86% (Cerebellum), 85.69% (Cingulo‐opercular), 76.27% (Fronto‐parietal), 72.94% (Occipital), and 90.20% (Sensorimotor). Logistic Regression yields accuracies ranging from 71.18% to 86.27%. Notably, the Sensorimotor, Cingulo‐Opercular and DMN networks display distinctive connectivity alterations in MCI, reflecting the versatility and generalizability as well as the medical correctness of our approach. These findings underscore the method's robust ability to discern intricate brain connectivity patterns and contribute to potential clinical relevance for early neurodegenerative disorder diagnosis and intervention

**Conclusion:**

Our study showcases the effectiveness of a graph‐theoretic approach in capturing the intricate connectivity dynamics of fMRI time‐series data across multiple networks. By representing each subject within a network through graphs, we unveil the subtleties of MCI pathology. The derived insights not only contribute to enhanced diagnostic precision but also shed light on the intricate relationships within brain networks.